# Actigraphy Meets AI: A Digital Biomarker for Parkinson's Disease and Isolated REM Sleep Behaviour Disorder

**DOI:** 10.1111/jsr.70396

**Published:** 2026-07-07

**Authors:** Leonor Lopes, Jan D. Warncke, Irina Filchenko, Kuangyu Shi, Claudio L. A. Bassetti, Carolin Schäfer

**Affiliations:** ^1^ Department of Nuclear Medicine, Inselspital, Bern University Hospital University of Bern Bern Switzerland; ^2^ Graduate School for Cellular and Biomedical Sciences University of Bern Bern Switzerland; ^3^ Schlaf‐Wach‐Epilepsie Zentrum, Department of Neurology, Inselspital, Bern University Hospital University of Bern Bern Switzerland

**Keywords:** actigraphy, artificial intelligence, iRBD, Parkinson's disease

## Abstract

Isolated rapid eye movement (REM) sleep behaviour disorder (RBD) is an early stage of alpha‐synucleinopathies, such as Parkinson's disease (PD). Actigraphy is a promising tool for assessing rest‐activity patterns, however its diagnostic or predictive value in these disorders is understudied. This study evaluated AI‐based models applied to actigraphy data to improve the detection of PD and RBD, additionally exploring their capability to capture early neurodegenerative patterns in isolated RBD. We trained and evaluated machine learning models with extracted actigraphy features, a convolutional neural network and a pre‐trained transformer model—PAT—on two classification tasks: (1) distinguishing patients with PD from non‐neurodegenerative controls and (2) distinguishing patients with PD and RBD (PD‐RBD) from those without (PD‐noRBD). We tested the best‐performing model on patients with isolated RBD. PD‐RBD showed significantly altered actigraphy features compared to PD‐noRBD and controls. In PD versus control classification, the PAT model performed best (AUC = 0.937, sensitivity = 80.5%, specificity = 92.9%) followed by the neural network (AUC = 0.863) and machine learning (AUC = 0.840). In the PD‐RBD versus PD‐noRBD classification task, the PAT model again outperformed the other models (AUC = 0.956, sensitivity = 84.4% and specificity = 92.9%). For both tasks, the scores of isolated RBD patients fell between those of the comparison groups. AI‐based actigraphy models can distinguish PD patients from controls and identify subtypes with high sensitivity and specificity. This shows their potential as non‐invasive, scalable diagnostic tools and their relevance for identifying prodromal motor features, with future applications in predicting isolated RBD progression to PD. Validation in longitudinal cohorts remains necessary.

## 1 Introduction

Parkinson's disease (PD) is the neurological disorder with the fastest increase in prevalence (Steinmetz et al. 2024), characterized by both motor symptoms, such as bradykinesia, rigidity and tremor, and non‐motor symptoms, including cognitive, sensory and autonomic dysfunction (Dauer and Przedborski 2003). Among the most common non‐motor symptoms of PD is REM sleep behaviour disorder (RBD), which affects approximately 50% of patients (Hu 2020).

RBD exists not only as a symptom of PD (secondary RBD) but also in an isolated form (iRBD). iRBD is recognized as an early stage of α‐synucleinopathies, the overt clinical forms which include PD, dementia with Lewy bodies (DLB) and multiple system atrophy (MSA) (Högl et al. 2018; Iranzo et al. 2006). The prevalence of iRBD is documented to be about 1% in individuals over 60 years old (Haba‐Rubio et al. 2018). Clinically, RBD is characterized by the enactment of vivid and often violent dreams (Sateia 2014). Polysomnography (PSG) records dream enactment episodes and REM sleep without muscle atonia (RSWA), both diagnostic criteria for RBD (Sateia 2014).

iRBD precedes the onset of overt clinical symptoms of α‐synucleinopathies by up to 20 years (Iranzo et al. 2016). The conversion rate to overt α‐synucleinopathies is about 6.3% per year (Postuma et al. 2019). The extended prodromal period represents a window for early diagnosis and possible future disease‐modifying intervention. However, there is currently no reliable and easily accessible biomarker to predict conversion from iRBD to an overt α‐synucleinopathy.

Actigraphy, a non‐invasive and easily accessible method for monitoring movement, can detect alterations in activity associated with neurodegenerative disorders (Pan et al. 2014; Smith et al. 2018). Few studies assessed its diagnostic potential in iRBD and PD. In one study, the number of wake bouts at night distinguished RBD in PD patients (PD‐RBD: *n* = 23; PD‐noRBD: *n* = 22) with high specificity (95.5%), though sensitivity remained low (20.1%) (Louter et al. 2014). Furthermore, machine learning (ML) classifiers which were trained on actigraphy data distinguished PD patients with and without RBD with high accuracy (92.9%), sensitivity (94.9%) and specificity (92.7%), however the sample size was limited (PD‐RBD: *n* = 18; PD‐noRBD: *n* = 8) (Raschellà et al. 2023). Reduced relative amplitude of actigraphy records, a non‐parametric circadian metric indicating a desynchronization between the main active and main rest periods, can differentiate iRBD from other sleep movement disorders and healthy controls (Raschellà et al. 2023), as well as nighttime and 24‐h rhythm features (Brink‐Kjaer, Winer, et al. 2023). Increased time in bed, sleep latency, and low sleep efficiency also distinguished iRBD from healthy controls (Liguori et al. 2021).

A recent study compared the actigraphy‐rest‐activity patterns of patients with secondary RBD, iRBD and controls, showing a progressive increase in probable napping behaviour, activity fragmentation and physical inactivity from controls to iRBD to overt α‐synucleinopathies (Feng et al. 2020; Hammad et al. 2021). Notably, the first comparison of actigraphy patterns between iRBD converters and non‐converters revealed that converters exhibited increased daytime napping and reduced daytime activity (Feng et al. 2020).

Despite these promising findings, several limitations remain unaddressed. Previous studies relied on small datasets, hand‐crafted features, or conventional statistical methods, limiting their potential to use all information from actigraphy data, thus limiting sensitivity to subtle disease‐related patterns. Furthermore, no study has attempted to integrate actigraphy into predictive models, with future applications in phenoconversion in iRBD.

Artificial intelligence (AI), particularly deep learning, offers a promising solution by automatically extracting complex temporal patterns from raw or minimally processed actigraphy data that may not be captured by conventional methods. Studies have shown reliable performance of AI algorithms in sleep staging (Boe et al. 2019; Walch et al. 2019), in diagnosing sleep disorders (Brink‐Kjaer, Gupta, et al. 2023; Kusmakar et al. 2021), as well as monitoring of neurodegenerative disorders (Cole et al. 2011; Bikias et al. 2021). However, the use of advanced AI models based on actigraphy in the classification of PD and its subtypes, and their application to predicting disease progression in iRBD, remains largely unexplored.

Our biological hypothesis is that iRBD patients exhibit subtle motor activity disruptions that resemble early‐stage PD and can be captured by actigraphy. Our methodological hypothesis is that AI models can learn informative temporal features from actigraphy signals that conventional methods miss, enabling better diagnostic classification and stratification of disease risk. Ultimately, this study represents a step toward combining AI models and actigraphy as an objective, accessible tool for early detection, stratification and longitudinal monitoring in neurodegenerative disorders.

## 2 Methods

### 2.1 Study Population

Our cohort consists of 45 PD patients with RBD (PD‐RBD), 42 PD patients without RBD (PD‐noRBD), 47 non‐neurodegenerative control patients with Restless Legs Syndrome, and 6 patients with isolated RBD.

Patients were included with a confirmed diagnosis of PD given by a specialist of the local centre of movement disorders (according to the Movement Disorder Society criteria for PD; Postuma et al. 2015). RBD, isolated or secondary, was diagnosed by a somnologist based on the ICSD III criteria (Sateia 2014) after PSG. Control participants with the diagnosis of Restless Legs Syndrome were diagnosed by a somnologist in the local centre based on the ICSD III criteria (Sateia 2014).

### 2.2 Study Design

Actigraphy records were collected retrospectively (from 01/01/2000 to 31/12/2020) from patients who either gave general consent for use of their pseudonymized data or provided written informed consent for further use of their data within the retrospective or prospective RBD studies which have been approved by the local ethical committee (BASEC 2022‐01379 and 2021‐00619). Records of patients were only included if the diagnosis of isolated RBD or secondary RBD was certain, and if symptomatic RBD provoked by medication or sleep‐disordered breathing was ruled out or deemed unlikely. Records were excluded if the RBD diagnosis was uncertain or potentially caused by medication or sleep‐disordered breathing.

Age, gender, disease duration, and UPDRS‐III scores were collected when available.

### 2.3 Data Acquisition and Preprocessing

Actigraphy datasets were available retrospectively from participants recruited under approved protocols. The recordings were obtained using the Motionlogger device (Ambulatory Monitoring, Ardsley, NY), operated in Zero Crossing mode with default settings. The device has a sampling rate of 10 Hz. However, the original high‐frequency data are not stored. Instead, the number of times the signal voltage crosses a predefined threshold is aggregated by the device into 1‐min epochs and stored as activity counts, representing movement frequency per minute. All subsequent analyses were performed using these 1‐min epoch activity time series. Movement count per epoch is further designated as activity. The first and last day of recordings were eliminated as they did not have the full day of activity data. For deep learning models, before inputting into the networks, five random days of the actigraphy record were selected and logarithmic normalization was applied. As recording durations varied across participants, five full days of actigraphy were selected for the main analysis to ensure a fixed input length while maximizing cohort retention (approximately 90%). Results with 3 and 7 days are also presented in Supporting Information.

### 2.4 Feature Extraction

Feature extraction captured key actigraphy‐derived characteristics, including statistical, rest‐activity, sleep‐related, frequency‐domain and night movement‐based features. Basic statistical features were computed, including mean, standard deviation, median, maximum, interquartile range, entropy, skewness, and kurtosis of activity values. Rest‐Activity features were derived using the PyActigraphy (Hammad et al. 2021) package 1.2.2, implemented in Python 3.10, namely total average daily activity (ADAT), interdaily stability (IS, quantifying the repeatability of the patters over each day), intradaily variability (IV, quantifying the variability of activity or rest/activity fragmentation), mean activity during the least 5 active hours (L5) and during the 10 most active hours (M10) and relative amplitude (RA, quantifying the RA between M10 and L5) (Van Someren et al. 1997; Witting et al. 1990). The total sleep time, sleep efficiency and wake after sleep onset were calculated using the Sadeh algorithm. Frequency‐domain features were extracted from the power spectral density of the activity signal using the Welch method with a Hann window (Welch 1967). Using spectral analysis, we quantified the strength of different rhythms (e.g., 24‐h or shorter cycles) by calculating the power at those frequencies. The dominant frequency, total power and power contributions of ultradian (i.e., 3–6 h cycles) (Thornton et al. 2024) were computed to assess periodic patterns in activity fluctuations. Both the sum of the power corresponding to those frequencies, the maximum power and the relative power (i.e., as divided by power of all frequencies) were extracted. Additionally, movement episodes during the night were analysed in the period between 22:00 and 6:00. Movement episodes were defined based on an adaptive activity threshold, set to one standard deviation above the mean. For each night, the number of movement episodes was recorded and the duration, peak amplitude, mean amplitude, time since the previous movement and time to the next movement were computed. The average number of movement episodes per night was also included.

### 2.5 Classification Strategies

Two classification tasks were performed: (1) Controls versus PD (including PD‐RBD and PD‐noRBD) and (2) PD‐RBD versus PD‐noRBD. To comprehensively evaluate diagnostic performance, we employed multiple classification approaches on both tasks:

#### 2.5.1 Machine Learning Models With Extracted Features

To classify actigraphy recordings, a set of ML models was trained using extracted time‐domain, frequency‐domain and circadian rhythm features. The models evaluated included logistic regression (LR), random forest (RF), support vector machine (SVM) and XGBoost (XGB). These classifiers were selected for their complementary strengths in handling structured feature sets and varying decision boundaries. Details on ML models are given on Supporting Information.

#### 2.5.2 Convolutional Neural Networks

A 1D Convolutional Neural Network (CNN) was implemented to learn discriminative patterns directly from the actigraphy signals for the classification of actigraphy data. The data were preprocessed by selecting random 5‐day segments for each sample and applying logarithmic normalization. The CNN was trained in an end‐to‐end manner, using the signal segments as input and the corresponding diagnostic labels (Controls vs. PD and PD‐RBD vs. PD‐noRBD) as targets. More details on the CNN architecture and training are given in the Supporting Information.

#### 2.5.3 Pretrained Actigraphy Transformer

The Pre‐trained Actigraphy Transformer (PAT) is a transformer‐based foundation model described in detail in Ruan et al. (2024), that was previously pre‐trained on a large corpus of actigraphy data (29,307 participants from the National Health and Nutrition Examination Survey; National Center for Health Statistics n.d.) in an unsupervised way. This pre‐trained model, which learned representations of actigraphy patterns, was fine‐tuned for our specific classification tasks. The data was normalized as previously described for the CNN model. Further details on the model and training are given in the Supporting Information.

### 2.6 Performance Evaluation

All models were trained and evaluated using leave‐one‐out cross‐validation, to ensure a more robust estimation of generalization performance. Classification outcomes were assessed using sensitivity, specificity, positive predictive value (PPV), negative predictive value (NPV) and area under the receiver operating characteristic curve (AUC‐ROC). For the primary analysis, thresholds for binary classification were selected using the Youden index, which maximizes the overall trade‐off between sensitivity and specificity. To assess potential applicability as a screening tool, model performance was additionally evaluated at thresholds corresponding to a sensitivity of 95%. In addition, confusion matrices were analysed to understand the patterns of misclassification. Feature importance analyses identified key actigraphy‐derived biomarkers contributing to classification performance across different ML methods. The best model was tested in a small iRBD test set (*n* = 6). The model scores for controls, iRBD and PD are shown and compared.

### 2.7 Statistical Analysis

All analyses were implemented in Python 3.12. The CNN model was built using PyTorch 2.6.0 and the PAT finetuning was performed with TensorFlow 2.18. Conventional ML models were implemented using scikit‐learn 1.6.1.

Statistical differences of descriptive statistics between groups were assessed either by chi‐square test of independence, for categorical variables or by *t*‐test or Mann–Whitney U test, for continuous variables. Shapiro–Wilk test was used to assess sample normality, and statistical tests were chosen accordingly. Statistical differences in the actigraphy‐derived features and model scores between groups were assessed using linear regression models including cohort (controls, PD‐RBD, PD‐noRBD) as a categorical predictor and age and gender as covariates. For each feature, we extracted the *p* values corresponding to group comparisons from the regression model. Age‐ and gender‐adjusted model scores and actigraphy variables were obtained by removing the estimated age and gender effects from each individual value and visualized using ROC curves and boxplots of corrected values. Two‐sided *p* values < 0.05 were considered significant.

## 3 Results

### 3.1 Subjects

Forty‐two PD‐noRBD (age: 62.0 ± 6.6 years [mean ± standard deviation], 21 male), 45 PD‐RBD (age: 65.2 ± 7.2 years, 28 male) and 47 control patients with restless legs syndrome (age: 61.4 ± 9.2 years, 35 male) were included in the analysis. Six additional iRBD patients (age: 65.3 ± 4.6, 6 male) were also included. Demographic information of all patients is presented in Table 1.

**TABLE 1:** Demographic data of all patients and subgroups.

	Controls (RLS)	PD without RBD	PD with RBD	iRBD
Number of subjects	47	42	45	6
Age	61.4 ± 9.2	62.0 ± 6.6	65.2 ± 7.2	65.3 ± 4.6
Gender (F/M)	12/35	21/21	17/28	0/6
UPDRS‐III	—	24.2 ± 10.0 (*n* = 38)	19.6 ± 9.3 (*n* = 28)	—
Disease duration (years)	—	9.6 ± 3.6 (*n* = 42)	11.9 ± 5.1 (*n* = 43)	—
Actigraphy duration (days)	9.8 + 3.5	8.4 ± 3.6	7.8 ± 3.8	10.2 + 3.8

*Note:* Continuous variables are shown as mean ± standard deviation (SD). Categorical variables are shown as count. Disease duration is calculated as the time of disease diagnosis to the actigraphy record date. When information is unavailable for the full sample, the number of patients with information available is presented in brackets.

There were significant differences in age (*p* = 0.043) and actigraphy record duration (*p* = 0.008), but not in gender between controls and all PD patients (PD with and without RBD). There were also significant differences in age (*p* = 0.036) and disease duration (*p* = 0.020) between PD with and PD without RBD, with no differences in gender, actigraphy record duration or Unified Parkinson Disease Rating Scale part III (UPDRS‐III).

### 3.2 Comparison of Classical Actigraphy Features

We started the analysis with the comparison of actigraphy features of PD‐RBD, PD‐noRBD and RLS controls with the aim of identifying distinct pattern differences in the respective groups.

Figure 1a shows the mean activity levels for each group across the 24‐h period. Overall, the activity profiles are largely overlapping between groups, with no major differences during the daytime. However, during the night, individuals with PD‐RBD exhibit higher activity levels compared to those with RLS and PD‐noRBD.

**FIGURE 1:**
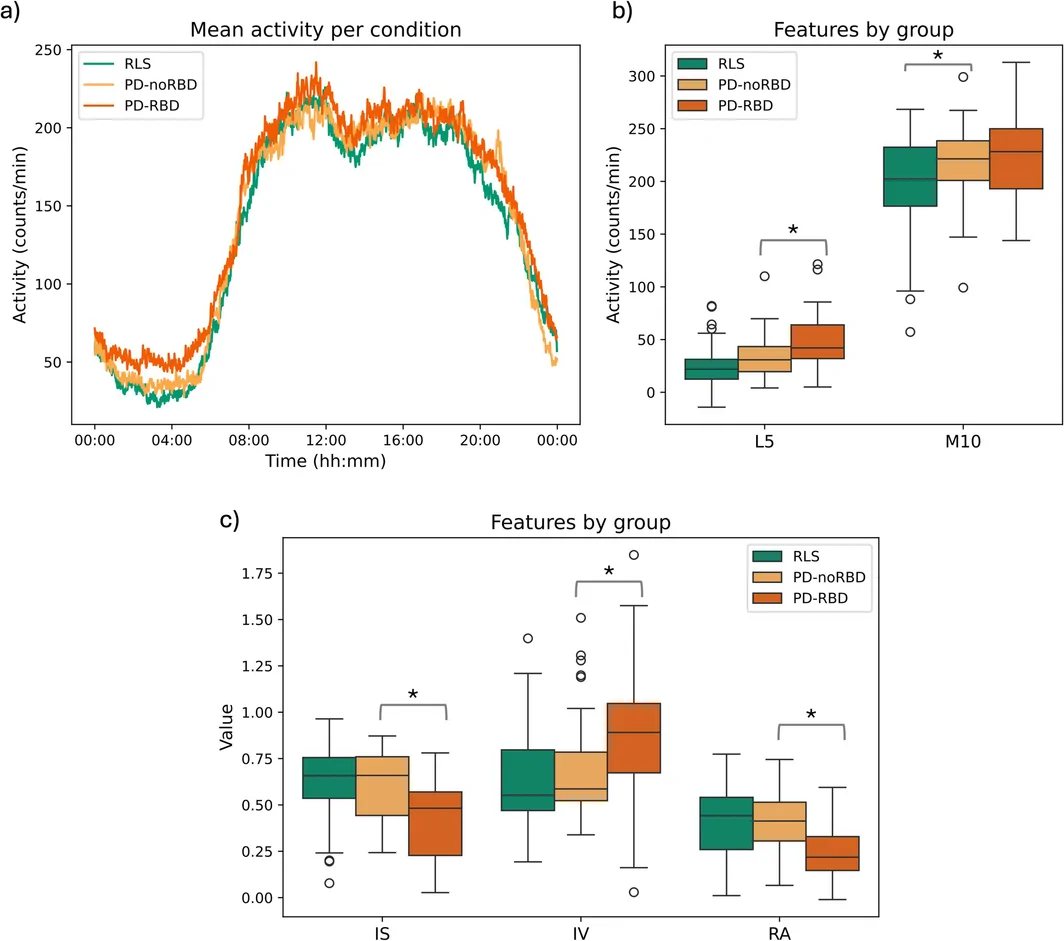
Actigraphy features per group. (a) Mean daily activity per group. Activity, in counts/min, is calculated as previously described in the Methods section. Time refers to the hours and minutes (hh:mm) of the 24‐h period. (b) Boxplots of the mean activity during the least 5 active hours (L5) and during the 10 most active hours (M10). (c) Boxplots of the interdaily stability (IS), intradaily variability (IV) and relative amplitude (RA). The *y*‐axis represents the value of each one of the variables represented. *p < 0.05.

In Figure 1b, significant differences were observed for several non‐parametric features. IS was significantly lower in PD‐RBD compared to both PD‐noRBD and RLS (*p* < 0.001), while no difference was found between PD‐noRBD and RLS (*p* = 0.679). IV was higher in PD‐RBD compared to PD‐noRBD (*p* = 0.006) and RLS (*p* = 0.002), whereas no difference was observed between PD‐noRBD and RLS (*p* = 0.718). RA was significantly reduced in PD‐RBD compared to both PD‐noRBD and RLS (*p* < 0.001), with no difference between PD‐noRBD and RLS (*p* = 0.695). Mean activity during the least 5 active hours was significantly higher in PD‐RBD compared to PD‐noRBD (*p* = 0.001) and RLS (*p* < 0.001), while PD‐noRBD and RLS showed no significant difference (*p* = 0.093). Mean activity during the 10 most active hours was significantly higher in PD‐noRBD (*p* = 0.007) and PD‐RBD (*p* = 0.003) than in RLS, with no differences between PD‐noRBD and PD‐RBD (*p* = 0.751).

### 3.3 AI‐Based Models—Controls Versus PD Classification

Since we hypothesized that AI outperforms the classical actigraphy features in distinguishing individuals with PD from controls, we further assessed the ability of actigraphy‐based AI models in this regard.

We tested three classification approaches: a traditional ML model, a simple deep learning model (CNN) and a pretrained transformer (PAT). This comparison aimed to determine whether deep learning models, particularly those pretrained on actigraphy data, offer performance advantages over standard ML methods. For this comparison, the chosen ML model was LR, as it had the best AUC. Results for other ML models are presented in Table S1.

The PAT model had the highest classification performance, achieving an AUC of 0.937, followed by CNN (AUC = 0.839) and ML (AUC = 0.837; Figure 2a). Additionally, the PAT model outperformed the other approaches in terms of specificity (95.7%), positive predictive value (PPV = 97.2%) and negative predictive value (NPV = 72.6%). It also exhibited high sensitivity (80.5%). The CNN model had an intermediate performance with a sensitivity of 69.0% and specificity of 91.5%, and a high positive predictive value (PPV = 93.7%). The ML model had the lowest specificity (83.0%) and sensitivity of 71.3% (Figure 2b).

**FIGURE 2:**
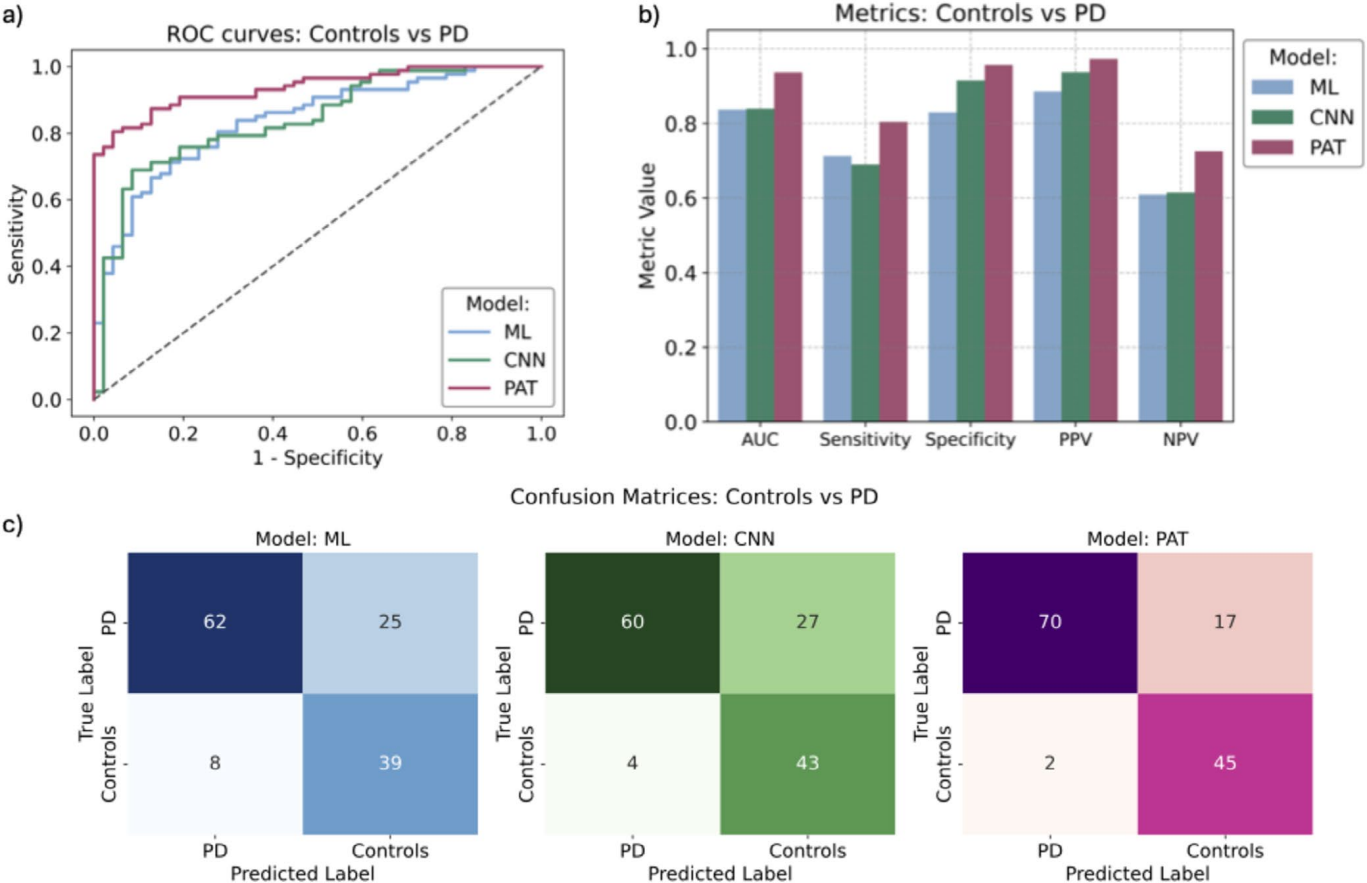
Classification performance of all methods in controls versus PD task. AUC—area under the ROC curve; CNN—convolution neural network; M—machine learning; NPV—negative predictive value; PAT—pre‐trained actigraphy transformer; PPV—positive predictive value.

The PAT model achieved the best balance between true positive and true negative classifications, with fewer misclassifications compared to ML and CNN. The CNN model had only 4 false positives but relatively high number of false negatives. The ML model demonstrated the similar performance, with strong specificity but struggled with false negatives, reflected in lower sensitivity (Figure 2c).

To identify the aspects of motor activity with the highest contribution to the classification of PD versus controls using actigraphy data, we next analysed feature importance within the best ML model. The top four most important features were standard deviation of activity, power at the dominant frequency (the strength of the most prominent rhythm, usually the 24 h cycle), L5 and ultradian power (strength of 3–6 h cycles). These features demonstrated coefficient values > 0.8, indicating their strong predictive capacity. Of lesser importance, but still contributing to classification, were ADAT, median activity, duration of night movements, total power and relative ultradian power (Figure 3a), all with coefficients 0.6–0.8.

**FIGURE 3:**
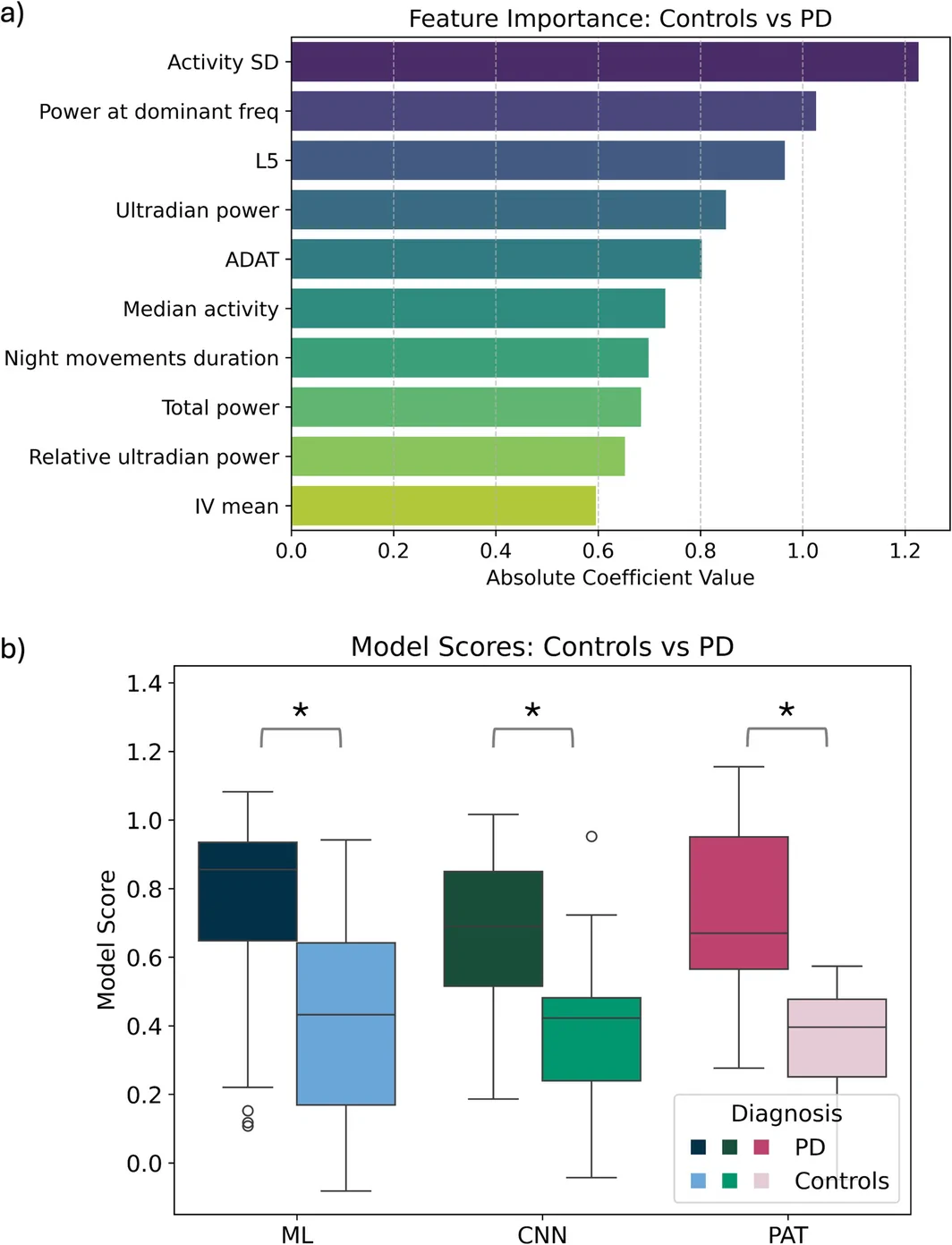
Model interpretability for controls versus PD task. ADAT—total average daily activity; freq—frequency; L5—mean activity during the least 5 active hours; SD—standard deviation. *p < 0.05 in the multivariate linear regression with age and gender.

To gain further insight into how each model distinguishes between PD and control subjects, we analysed the distribution of output scores generated by the ML, CNN and PAT models. This comparison assessed not only classification performance but also the confidence and consistency of predictions across groups. Figure 3b presents the model output scores per condition (Controls—lighter; PD—darker). In the ML model, PD predictions show a tight interquartile range compared to the broader distribution for controls, with good differentiation between groups (*p* < 0.001). The CNN model exhibited similar differentiation between groups (*p* < 0.001) but with wider interquartile ranges for PD and tighter for controls. The PAT approach showed the most pronounced difference between PD and controls scores (*p* < 0.001), consistent with the performance results. Interestingly, the interquartile range in the PD group is the highest and in the control group, the lowest. For both deep learning models (CNN and PAT), there is a relatively large interquartile range for the PD, and a shorter one for controls.

### 3.4 AI‐Based Models—Classification PD‐RBD Versus PD‐noRBD

We further explored the ability of the same AI approach to distinguish between PD with and without RBD actigraphy data using the previous workflow (see above). For this classification task, the best ML model was the LR, later defined as the ML method in this section. Comparisons with other ML models are shown in Table S2.

The classification performance of ML, CNN and PAT models in distinguishing PD‐RBD from PD‐noRBD is shown in Figure 4. Namely, in Figure 4a, the ROC curves are displayed, showing that the PAT model demonstrated superior classification performance with an AUC of 0.956, followed by CNN (AUC = 0.768) and ML (AUC = 0.672).

**FIGURE 4:**
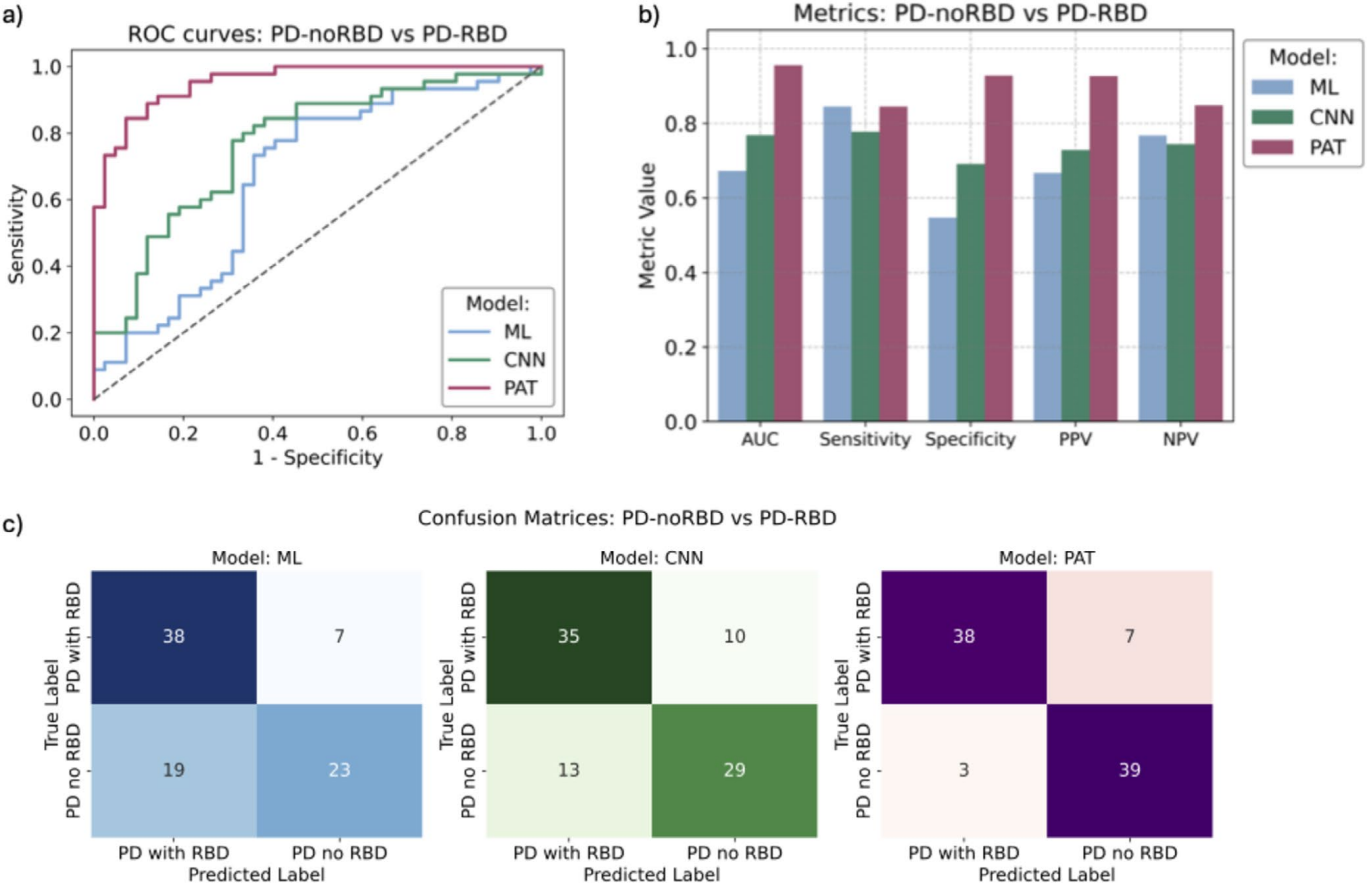
Classification performance of all methods in PD‐noRBD versus PD‐RBD task. AUC—area under the ROC curve; CNN—convolution neural network; ML—machine learning; NPV—negative predictive value; PAT—pre‐trained actigraphy transformer; PPV—positive predictive value.

As shown in Figure 4b, the PAT model achieved excellent specificity (92.9%) and sensitivity (84.4%). It also had the highest positive predictive value (PPV = 92.7%) and negative predictive value (NPV = 84.8%). The CNN model showed moderate performance (AUC = 0.768), with balanced sensitivity (77.8%) and specificity (69.0%). The ML model had the lowest overall performance (AUC = 0.672), with high sensitivity (84.4%) and low specificity (54.8%).

In Figure 4c, confusion matrices of the performance of each model are shown.

The PAT model exhibited the best classification balance, with only three misclassified PD‐noRBD and seven PD‐RBD. The ML model had less false negatives and more false positives than the CNN, corresponding to the best sensitivity but worse specificity.

The feature importance for the ML model was analysed and shown in Figure 5a. The most important features were the night movements maximum amplitude and the RA, showing absolute coefficient values higher than 1.2. Additional important features included the mean number of night movements, L5, duration of night movements and ADAT, all with coefficient values > 0.6.

**FIGURE 5:**
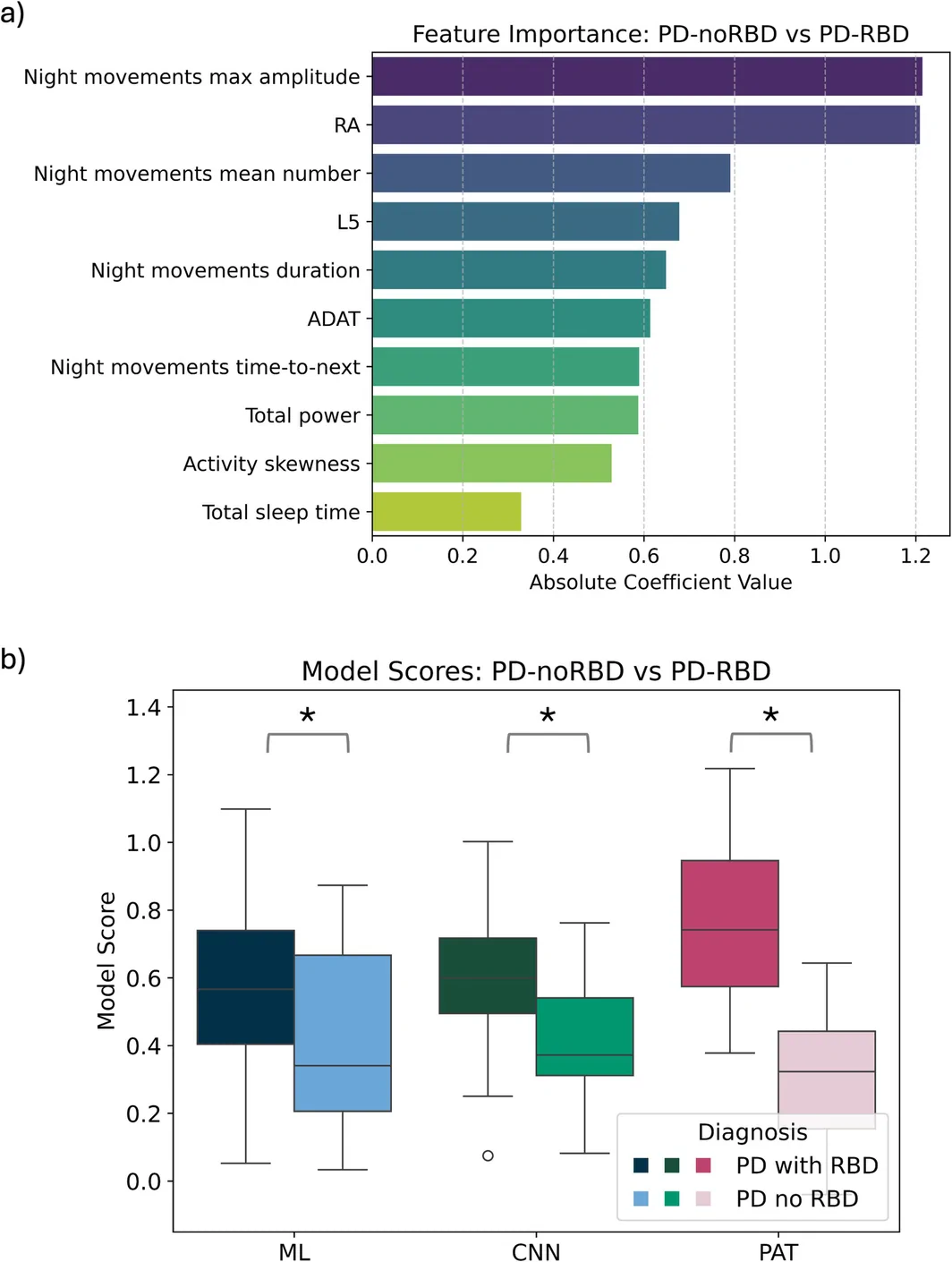
Model interpretability for PD‐noRBD vs PD‐RBD task. IQR—interquartile range; RA—relative amplitude; SD—standard deviation. *p < 0.05 in the multivariate linear regression with age and sex. *p < 0.05.

Figure 5b demonstrates the discriminatory capacity of the three classification methods scores (all *p* < 0.01) in distinguishing PD‐RBD (darker plots in the figure) from PD‐noRBD patients (lighter plots). Of notice is the relatively large interquartile range in the ML method in the PD‐noRBD group, the shorter interquartile ranges for the CNN model in both groups, and the highest for the PD‐RBD group in the PAT model. The PAT approach demonstrated the most pronounced difference between the groups, consistent with the high classification performance.

To further investigate which time intervals contributed to PAT‐based classification, we performed an attention‐based analysis of representative samples from each class. Higher attention weights were consistently observed during nocturnal periods, particularly in PD‐RBD cases, as well as during early afternoon intervals. Representative attention maps for each class are shown in Figure S12.

### 3.5 AI‐Based Models—iRBD Testing

To assess how individuals with iRBD are positioned along the learned group‐specific discrimination axes, we applied the best‐performing PAT models to the iRBD cohort and examined their output scores. In the PAT model trained with controls versus PD, the model output scores increase from controls to iRBD (*p* = 0.068) to PD (*p* = 0.003), as displayed in Figure 6a. In the PAT model trained for PD‐noRBD versus PD‐RBD differentiation, the model output scores increased significantly from PD‐noRBD to iRBD (*p* = 0.028) to PD‐RBD (*p* < 0.001).

**FIGURE 6:**
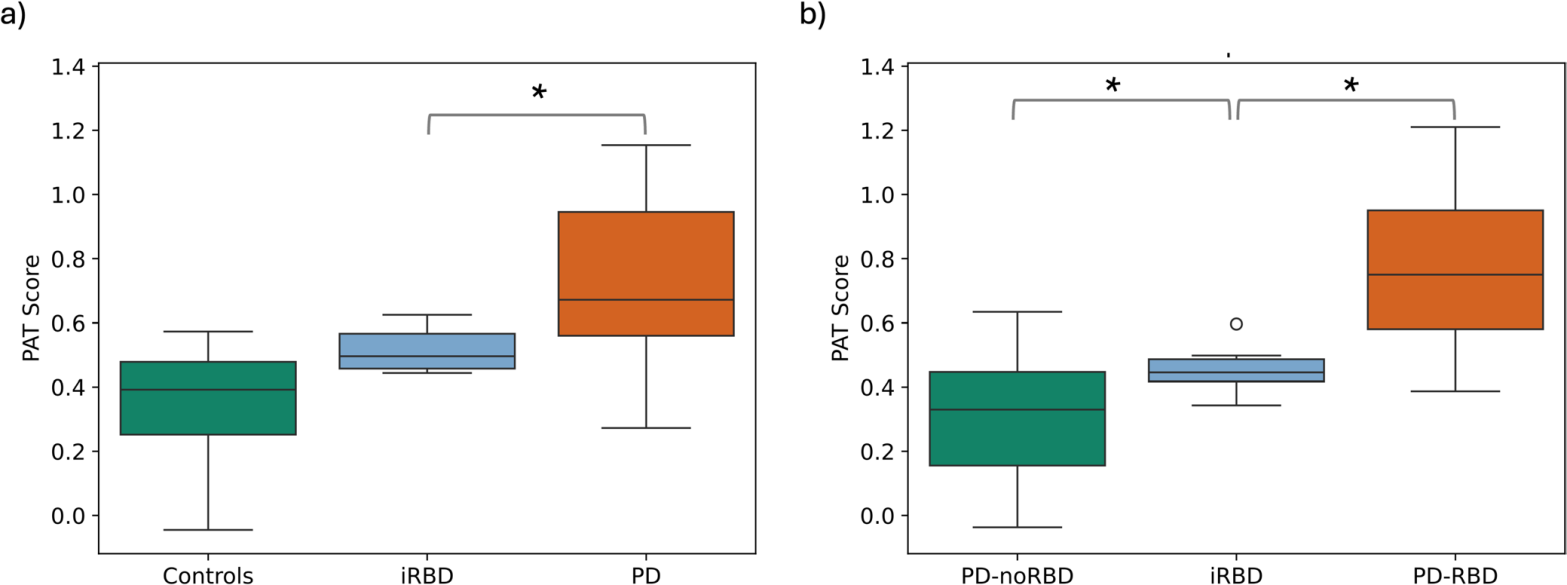
PAT model scores for the six iRBD patients for the classification task of (a) controls versus PD and (b) PD‐noRBD versus PD‐RBD. *p < 0.05.

## 4 Discussion

This study demonstrated the potential of actigraphy‐based AI models as non‐invasive digital tools for detecting PD and RBD. The pre‐trained transformer model, PAT, achieved high sensitivity and specificity in distinguishing PD from controls and in detecting RBD in PD patients. Furthermore, the classification scores of iRBD patients suggest that actigraphy‐based AI methods may capture prodromal activity patterns that differ from PD and control groups, with potential future use in predicting phenoconversion of iRBD to overt alpha‐synucleinopathies like PD.

We found that several non‐parametric rest‐activity actigraphy features significantly differed between PD patients with and without RBD. Specifically, the PD‐RBD group had higher IV (reflecting increased fragmentation of activity, including more daytime naps and nighttime movements), higher L5 values (indicating greater activity during the least active five‐hour period, typically at night) and lower IS (suggesting less consistency in daily rhythms) and RA (suggesting reduced contrast between active and rest periods, likely driven by increased nocturnal activity). These findings align with previous studies reporting higher nocturnal activity, higher fragmentation, lower sleep efficiency and more frequent napping in iRBD compared to controls (Feng et al. 2020; Filardi et al. 2020) and more nocturnal wake bouts in PD‐RBD than PD‐noRBD (Louter et al. 2014). Although Feng et al. (2020), showed lower physical activity during active periods from controls to iRBD, our M10 parameter was not significant which may indicate that only including the 10 most active hours can be less accurate.

Interestingly, we observed no significant differences in actigraphy features between PD‐noRBD and the control group. This is consistent with prior literature reporting minimal differences in sleep and rest‐activity parameters between healthy individuals and PD (Zhong et al. 2013). Nevertheless, other studies showed differences in IS and RA, as well as a phase advance in sleep onset and offset (Madrid‐Navarro et al. 2019). However, none of these studies evaluated the presence of RBD in the PD patients, limiting their interpretability as the contribution of each type is unknown. Additionally, our control group consisted of patients with RLS, a condition associated with sleep–wake disturbances, which may have attenuated observed differences. In summary, the observed differences in rest‐activity patterns between PD patients with and without RBD are consistent with the hypothesis of distinct PD subtypes, such as the proposed brain‐first and body‐first progression pathways (Xu et al. 2024), in which PD‐RBD has been associated with the body‐first subtype. However, actigraphy‐based findings alone cannot establish differences in underlying pathological propagation mechanisms. Our results should therefore be interpreted as consistent with, rather than directly supporting this hypothesis.

Our results confirm that actigraphy effectively differentiates PD patients from controls when combined with ML or DL methods. Among these, the PAT model demonstrated the highest performance (AUC = 0.937), outperforming both CNNs and traditional ML classifiers. While PD diagnosis is typically straightforward in later stages, the high accuracy of our PAT model using only actigraphy features without underlying clinical data is notable. Differentiating PD from atypical parkinsonian syndromes such as MSA or progressive supranuclear palsy (PSP) remains a clinically challenging and high‐impact application for future studies, although our dataset did not include samples from these conditions.

The superior performance of the PAT model suggests that feature extraction from activity counts may capture subtle movement pattern changes not fully represented by predefined actigraphy metrics. Additionally, using an already pre‐trained AI model (the PAT model) in actigraphy data may allow for easier extraction of relevant features. A downside is the lack of clear interpretation of the features relevant for classification. In this matter, the ML models can be an advantage. Analysis of feature importance in the ML model for distinguishing PD from controls identified standard deviation of activity, total power, L5, power at the dominant frequency and ultradian power as key discriminative features. These features are consistent with known manifestations of PD, such as lower sleep efficiency, more awakenings, increased daytime sleepiness and disrupted circadian rhythmicity, compared to healthy individuals (Obayashi et al. 2021; Peeraully et al. 2012).

Specifically, L5 alterations can reflect disrupted or fragmented sleep patterns and reduced nocturnal restfulness in PD (Peeraully et al. 2012). Increased standard deviation of activity may capture greater variability in motor activity due to sleep fragmentation or daytime sleepiness, consistent with higher scores on the Insomnia Severity Index and Epworth Sleepiness Scale reported in PD (Yong et al. 2011). Additionally, alterations in the total power and power at the dominant frequency may reflect disturbances in circadian rhythmicity or rest‐activity cycles, which are commonly altered in PD (Obayashi et al. 2021).

Clinically, distinguishing PD‐RBD from those without is highly relevant, given the association of secondary RBD with a more severe PD progression phenotype, with faster progression and increased cognitive decline (Liu et al. 2021; Nomura et al. 2013). The PAT model consistently achieved the best performance in this task (AUC = 0.956). ML feature analysis identified RA, interquartile range of the activity and mean number of night movements as important features, reinforcing the role of circadian rhythm disruptions in PD‐RBD. Additionally, attention‐based analysis of the PAT model indicated that classification decisions were primarily driven by activity patterns occurring during night periods and, to a lesser extent, early afternoon intervals. These temporal segments may correspond to increased night‐time motor activity and daytime napping behaviour. These findings support previous studies indicating that RBD patients have increased night‐time movement and disrupted rest‐activity rhythms. In fact, few studies showed that iRBD is characterized by the imbalance between nocturnal and diurnal motor activity (I < O index), reduced RA, increased time in bed and sleep latency and decreased sleep efficiency, compared to other sleep–wake disorders and controls (Louter et al. 2014; Bramich et al. 2022). The strong performance of our models suggests that actigraphy is a viable tool for identifying RBD in PD. As a non‐invasive, cost‐effective and scalable alternative, it can serve as a primary screening tool to identify high‐risk individuals who require confirmatory PSG. This approach reduces the burden on sleep laboratories and facilitates earlier clinical intervention. Furthermore, actigraphy enables the longitudinal monitoring of rest‐activity patterns over extended periods, which is not feasible with PSG. To prioritize sensitivity for screening contexts, the sensitivity‐specificity trade‐off can be adjusted through threshold selection, thus minimizing missed RBD cases. Our results show that even in thresholds with high sensitivity the PAT model still achieves good specificity (see Supporting Information).

To better understand the sources of misclassification, we conducted an additional analysis of the most challenging cases, defined as samples with the greatest disagreement between predicted and true labels. Qualitative inspection of their actigraphy records revealed atypical rest‐activity profiles relative to typical patterns of their respective groups. Quantitative analysis of actigraphy‐derived features in these cases further indicated deviations from group‐level distributions, indicating partial overlap in behavioural activity signatures. Representative examples are provided in Supporting Information.

Given the variability in recording duration inherent to retrospective actigraphy data, we performed supplementary analyses using different recording lengths (3, 5 and 7 days). Model performance was comparable across durations in both classification tasks, with only minor fluctuations in AUC (≤ 0.02–0.03). These findings suggest that the classification results were not strongly dependent on the selected recording length within this range (Figure S5).

### 4.1 Exploratory Analysis of iRBD Data

An exploratory component of our study was the evaluation of iRBD patients using the pre‐trained PAT model across two classification tasks: PD vs. controls and PD‐RBD vs. PD‐noRBD.

In the PD vs. control task, iRBD patients exhibited intermediate scores, increasing from controls to iRBD to PD. Although this positioning may reflect partial similarity of iRBD rest‐activity patterns to those observed in PD, it should be interpreted with caution, as the model was trained to distinguish PD from a clinical sleep‐disorder cohort rather than to capture disease progression. Accordingly, intermediate scores may reflect shared characteristics with both PD and RLS patterns rather than a true disease continuum. Nevertheless, the continuous model output may still indicate proximity to PD‐like rest‐activity signatures, which could hold relevance for risk stratification even without training with explicit progression labels.

A similar strategy has been applied in neuroimaging studies, where models trained to discriminate diagnostic groups were subsequently used to derive continuous disease‐relevant scores associated with phenoconversion risk or progression (Lopes et al. 2023; Lin et al. 2018). Prior actigraphy studies have shown that alterations in rest‐activity patterns (such as increased daytime napping and decreased activity levels) are associated with phenoconversion risk in iRBD (Feng et al. 2020), suggesting that such motor activity signatures may precede overt clinical symptoms. However, unlike these longitudinal imaging studies, our actigraphy dataset is cross‐sectional and lacks follow‐up conversion data. Therefore, the observed positioning of iRBD patients should be considered exploratory and hypothesis‐generating rather than indicative of individual conversion trajectories.

In the PD‐RBD versus PD‐noRBD task, iRBD scores again fell between the two PD groups, potentially reflecting variability in RBD‐related motor activity patterns. Integrating both classification approaches and data from other synucleinopathies may further refine understanding of iRBD conversion trajectories. However, longitudinal studies will be required to determine if these scores can eventually serve as robust markers for the risk or timing of conversion, comparable to imaging‐based biomarkers.

### 4.2 Strengths and Limitations

This study has several strengths. We performed a comprehensive analysis of actigraphy features and employed multiple classification frameworks—including ML, CNNs and the state‐of‐the‐art PAT transformer—allowing for systematic comparison across modelling approaches. The inclusion of distinct PD subtypes and iRBD patients supports the generalizability of our findings.

Several limitations should be acknowledged. The sample size, especially for iRBD, was small, limiting the robustness of predictive analyses. Nevertheless, given the low prevalence of iRBD in the general population and the challenges associated with its diagnosis and recruitment, the dataset still represents a valuable contribution. The use of RLS patients as controls is suboptimal, as RLS can independently affect sleep and motor activity patterns.

Additionally, PD patients were retrospectively recruited, and detailed, standardized information regarding sleep comorbidities (e.g., sleep‐disordered breathing), non‐sleep medical conditions and medication use (including dopaminergic therapy) was not consistently available for all participants. These factors may influence actigraphy‐derived metrics such as sleep duration (Yong et al. 2011) and nocturnal awakenings (Peeraully et al. 2012), and could therefore affect model performance. Although direct adjustment for these variables, we mitigated potential bias through statistical corrections for age and sex and the use of leave‐one‐out cross‐validation.

Furthermore, models were trained on device‐processed activity counts rather than raw acceleration signals, introducing potential device dependency due to differences in sensor characteristics and proprietary preprocessing across actigraphy systems. The extent to which the trained models would generalize to data acquired with other devices therefore remains uncertain. While leave‐one‐out cross‐validation provides an estimate of internal generalization, the absence of an independent external or out‐of‐distribution test set is an additional limitation. Multi‐centre external validation across different actigraphy devices will be essential to establish robustness and clinical applicability.

Although actigraphy captures motor activity patterns, it does not directly assess other prodromal PD symptoms such as autonomic dysfunction or cognitive impairment. Integrating actigraphy with other biomarkers may enhance its predictive accuracy.

Finally, although the PAT model demonstrated superior classification performance, its predictions are derived from latent temporal representations of minimally processed actigraphy signals that do not directly map onto clinically interpretable circadian or sleep‐related metrics. In contrast, ML models operate on predefined actigraphy features, allowing a more direct link to established physiological parameters. Further work should therefore focus on improving the explainability of transformer‐based approaches and validating their clinical applicability, especially in independent longitudinal cohorts.

## 5 Conclusion

This study demonstrates that actigraphy analysis, when combined with ML and deep learning approaches, can effectively distinguish between PD and control groups, and between PD patients with and without RBD. These findings support the potential of actigraphy as a non‐invasive digital biomarker for disease classification. Actigraphy‐based PAT model was further able to situate the iRBD data between controls and clinically diagnosed PD‐patients suggesting a potential role of actigraphy‐based classification in the early identification of individuals at increased risk of PD conversion. Future studies in larger, longitudinal cohorts with phenoconversion data are required to validate these findings and determine their prognostic utility.

## Author Contributions

**Jan D. Warncke:** methodology, writing – review and editing, visualization, validation, investigation. **Leonor Lopes:** methodology, conceptualization, software, data curation, formal analysis, validation, visualization, writing – review and editing, writing – original draft, investigation. **Claudio L. A. Bassetti:** conceptualization, writing – review and editing, supervision, resources, project administration, funding acquisition. **Irina Filchenko:** investigation, methodology, validation, writing – review and editing, visualization. **Carolin Schäfer:** conceptualization, investigation, writing – review and editing, funding acquisition, project administration, supervision, resources, data curation, visualization, methodology, writing – original draft. **Kuangyu Shi:** conceptualization, writing – review and editing, supervision, resources, project administration, funding acquisition.

## Funding

The authors have nothing to report.

## Conflicts of Interest

The authors declare no conflicts of interest.

## Supporting information


**Figure S1:** Schematic overview of the actigraphy‐based classification approaches. (a) Logistic regression model, as it was the used ML model in the main study results; (b) CNN used directly on the actigraphy records; (c) PAT Model with actigraphy records as input. ADAT—total average daily activity; CNN—convolutional neural network; ML—Machine Learning; mov—movements, L5—least 5 active hours; IS—interdaily stability; Q—Query; K—Key; V—Value.
**Figure S2:** Example case of RLS.
**Figure S3:** Example case of PD without RBD.
**Figure S4:** Example case of PD with RBD.
**Table S1:** Performance of ML models of the controls vs PD classification task. AUC: Area under the ROC curve; PPV: positive predictive value; NPV: negative predictive value; SVM: Support Vector Machine.
**Table S2:** Performance of ML models of the PD‐RBD versus PD‐noRBD classification task. AUC: Area under the ROC curve; PPV: positive predictive value; NPV: negative predictive value; SVM: Support Vector Machine.
**Figure S5:** ROC curves of the PAT model prediction using 3, 5 or 7 days of input actigraphy duration for both (a) controls versus PD and (b) PD‐noRBD versus PD‐RBD tasks.
**Figure S6:** Three RLS cases that were the hardest for the PAT model classification. The 2 first days were chosen for visualization.
**Figure S7:** Distribution of actigraphy‐derived features in problematic cases with Restless Legs Syndrome (RLS). Coloured dots represent individual cases identified as problematic by the classification model. Visual comparison of individual cases against the overall feature distributions highlights deviations in each metric that may contribute to model misclassification. Metrics where problematic cases had the greatest deviation from the median were chosen to display.
**Figure S8:** Three PD cases without RBD that were the hardest for the PAT model classification. The 2 first days were chosen for visualization.
**Figure S9:** Distribution of actigraphy‐derived features in problematic cases with PD without RBD. Coloured dots represent individual cases identified as problematic by the classification model. Visual comparison of individual cases against the overall feature distributions highlights deviations in each metric that may contribute to model misclassification. Metrics where problematic cases had the greatest deviation from the median were chosen to display.
**Figure S10:** Three PD cases with RBD that were the hardest for the PAT model classification. The 2 first days were chosen for visualization.
**Figure S11:** Distribution of actigraphy‐derived features in problematic cases with PD with RBD. Coloured dots represent individual cases identified as problematic by the classification model. Visual comparison of individual cases against the overall feature distributions highlights deviations in each metric that may contribute to model misclassification. Metrics where problematic cases had the greatest deviation from the median were chosen to display.
**Figure S12:** PAT model attention maps over 24‐h actigraphy profiles across diagnostic groups. Representative 24‐h mean activity profiles (black line; counts/min) from (a) RLS, (b) PD without RBD and (c) PD with RBD. The heatmap reflects the relative importance assigned by the model to different time points during classification—RLS versus PD in (a) and PD‐RBD versus PD‐noRBD in (b) and (c)—based on its attention scores. These scores represent how strongly each segment of the 24‐h signal contributed to the model's prediction. Values were normalized and projected onto a colour scale, where red indicates time periods with greater influence on the decision and blue indicates lower influence.

## Data Availability

The datasets generated and/or analysed during the current study are available from the corresponding author upon reasonable request. The code is available for download at: https://github.com/lopes‐leonor/ActiAI.
