# Supplementary material for: Actigraphy Meets AI: A Digital Biomarker for Parkinson's Disease and Isolated REM Sleep Behaviour Disorder
Source: J Sleep Res. 2026 Jul 7;35(5):e70396. doi: 10.1111/jsr.70396 (PMC13619045; doi:10.1111/jsr.70396)
Supplement: Supplementary file 1 [file JSR-35-e70396-s001.docx]

**Supplementary data**

**AI model details**

A schematic representation of the 3 models used in this study is presented in supplementary figure 1.

**Supplementary Figure 1.** Schematic overview of the actigraphy-based classification approaches. a) Logistic regression model, as it was the used ML model in the main study results; b) CNN used directly on the actigraphy records; c) PAT Model with actigraphy records as input. Abbreviations: ADAT – total average daily activity; CNN – convolutional neural network; ML – Machine Learning; mov – movements, L5 – least 5 active hours; IS - interdaily stability; Q – Query; K – Key; V – Value.

**Machine Learning**

The models evaluated included logistic regression, random forest, support vector machine, and XGBoost. Logistic Regression was implemented as a baseline model, employing an L2 regularization penalty to prevent overfitting. The optimization was performed using a L-BFGS solver. Random Forest, an ensemble-based decision tree model, was trained using 100 estimators, with each tree constructed using a bootstrap sample of the data. The Gini impurity criterion was applied to optimize node splitting. Support Vector Machine (SVM) was trained using a linear kernel, with regularization parameter set to 1. XGBoost, a gradient-boosted decision tree algorithm optimized, was implemented with a log-loss as evaluation metric.

**Convolutional Neural Networks**

The convolutional neural network (CNN) model consisted of three convolutional blocks followed by a global average pooling layer and a last fully connected classifier layer. Each convolutional block included a one-dimensional convolutional layer, instance normalization, a ReLU activation function, and dropout. The first convolutional layer had 128 filters with a kernel size of 8, the second had 256 filters with a kernel size of 5, and the third had 128 filters with a kernel size of 3. A global average pooling layer was applied to reduce the feature maps before passing them to a fully connected layer with softmax activation for classification. Kaiming uniform initialization was used for weight initialization.

The CNN was trained using a batch size of 16 for up to 500 epochs with an initial learning rate of 0.0004 (for controls versus PD) and 0.002 (for PD-RBD versus PD-noRBD), as determined by hyperparameter tunning. The Adam optimizer was used with weight decay set to 0.0014 (for controls versus PD) and 0.0008 (for PD-RBD versus PD-noRBD), and early stopping with a patience of 100 epochs was implemented to avoid overfitting.

**Pretrained Actigraphy Transformer**

The pre-trained actigraphy transformer (PAT) model was previously pre-trained on a large corpus of actigraphy data, learning representations of actigraphy patterns. We fine-tuned it for our specific classification tasks by attaching a task-specific classification head. The classification head consisted of a Global Average Pooling layer, a dropout layer and 2 fully connected linear layers with a final sigmoid activation. Fine-tuning was performed by optimizing the entire network on the labelled data, thus adapting the rich, pre-trained features to the nuances of our diagnostic categories. The smaller PAT model was chosen and trained for 500 epochs with a learning rate of 5.33 x 10^-6^, a batch size of 4 and Adam optimizer. The dropout rate was 0.5. Early stop was applied if validation loss did not improve after 100 epochs.

**Example of actigraphy records for each group**

To illustrate typical rest–activity patterns observed in each group, we provide representative examples of five consecutive days of actigraphy recordings from one participant in each cohort: RLS controls (Supplementary Figure 2), PD without RBD (Supplementary Figure 3), and PD with RBD (Supplementary Figure 4).

Supplementary Figure 2. Example case of RLS.

Supplementary Figure 3. Example case of PD without RBD.

Supplementary Figure 4. Example case of PD with RBD.

**Machine Learning models for Controls versus PD classification**

**Supplementary Table 1. Performance of ML models of the controls vs PD classification task.** AUC: Area under the ROC curve; PPV: positive predictive value; NPV: negative predictive value; SVM: Support Vector Machine.

| **Model** | **AUC** | **Sensitivity** | **Specificity** | **PPV** | **NPV** |
| --- | --- | --- | --- | --- | --- |
| **Logistic Regression** | **0.837** | **0.713** | **0.830** | **0.886** | **0.609** |
| **SVM** | 0.768 | 0.529 | 0.894 | 0.902 | 0.506 |
| **Random Forest** | 0.799 | 0.828 | 0.745 | 0.857 | 0.700 |
| **XGBoost** | 0.761 | 0.793 | 0.702 | 0.831 | 0.647 |

**Machine Learning models for PD-RBD versus PD-noRBD classification**

**Supplementary Table 2. Performance of ML models of the PD-RBD vs PD-noRBD classification task.** AUC: Area under the ROC curve; PPV: positive predictive value; NPV: negative predictive value; SVM: Support Vector Machine.

| **Model** | **AUC** | **Sensitivity** | **Specificity** | **PPV** | **NPV** |
| --- | --- | --- | --- | --- | --- |
| **Logistic Regression** | **0.672** | **0.844** | **0.548** | **0.667** | **0.767** |
| **SVM** | 0.652 | 0.600 | 0.714 | 0.692 | 0.625 |
| **Random Forest** | 0.655 | 0.689 | 0.643 | 0.674 | 0.659 |
| **XGBoost** | 0.637 | 0.867 | 0.405 | 0.609 | 0.739 |

**PAT model ROC curves with different input actigraphy record duration**

We also tested the PAT model using different durations of the actigraphy records that were input to the model. We present here the ROC curves for both classification tasks using 3 (PD-RBD n=48; PD-noRBD n=49; RLS n=49), 5 (PD-RBD n=45; PD-noRBD n=42; RLS n=47), and 7 (PD-RBD n=17; PD-noRBD n=23; RLS n=30). Performance was comparable across durations in both classification tasks, with only small fluctuations in AUC (≤0.02-0.03). Interestingly, the optimal duration was not identical across tasks: PD vs control classification showed similar or slightly higher performance with shorter segments, whereas PD-RBD vs PD-noRBD classification was highest at 5 days. This likely reflects task-dependent temporal characteristics of the discriminative signal. Distinguishing PD from controls may rely more strongly on stable global rest–activity rhythm differences that are captured within a few days, whereas distinguishing PD subtypes with and without RBD may benefit from additional nights to better capture variability in nocturnal motor patterns.

Supplementary Figure 5. ROC curves of the PAT model prediction using 3, 5 or 7 days of input actigraphy duration for both a) controls vs PD and b) PD-noRBD vs PD-RBD tasks.

**PAT model performance metrics optimized for screening**

To evaluate the potential applicability of the proposed models in a screening-oriented setting, we assessed classification performance at decision thresholds corresponding to a fixed sensitivity of approximately 95%. These thresholds were derived from the receiver operating characteristic (ROC) curves for each model.

In the PD-RBD vs PD-noRBD classification task, the thresholds required to achieve around 95% sensitivity were 0.174 for the ML model, 0.311 for the CNN model, and 0.452 for the PAT model. At these thresholds, the ML, CNN, and PAT models achieved sensitivities of 95.6%, with corresponding specificities of 14.3%, 26.2%, and 78.6%, respectively.

In the RLS vs PD classification task, the thresholds required to achieve 95% sensitivity were 0.225 for the ML model, 0.351 for the CNN model, and 0.409 for the PAT model. At these thresholds, the ML, CNN, and PAT models achieved sensitivities of 95.4%, with corresponding specificities of 29.8%, 40.4%, and 55.3%, respectively.

These results illustrate that model operating characteristics can be adjusted according to clinical requirements. In particular, the PAT model retained substantially higher specificity compared to ML and CNN approaches at equivalent sensitivity levels in both classification tasks.

**Problematic Cases**

In supplementary figure 6, we present problematic cases of RLS. These had some of the highest model output scores in PAT model of RLS vs PD classification. In supplementary figure 8 and 10 we present problematic cases of PD-noRBD and PD-RBD, respectively. These had model output scores that were closer to the opposite class. The distributions of the actigraphy-derived features in which these problematic cases most deviated from the cohort median are shown in supplementary figures 7, 9, and 11, highlighting potential sources of misclassification and reflecting atypical sleep and activity patterns relative to the overall population.

No specific metric was consistently altered in all problematic cases. For RLS, some problematic cases had higher levels of total daily activity and less total sleep time and sleep efficiency, typical of PD and thus why the model might have misclassified them. In supplementary figure 6, there seems to be more awakenings during the night and also increased daytime sleeping. Some cases also had lower skewness and kurtosis, indicating that the difference between low nighttime activity and high daytime activity is less pronounced, fewer distinct periods of very low activity (deep rest), and more frequent movement occurring throughout the night instead of being concentrated during wake periods. This results in fewer extreme activity values and reduced separation between rest and movement states.

For PD without RBD, the problematic cases were characterized by higher intradaily variability and lower interdaily stability, reflecting increased fragmentation of rest-activity rhythms, as well as higher number and magnitude of night movements, all more characteristic of PD with RBD. In supplementary figure 8, specially case #2, you can also notice the higher activity values during the night.

For PD with RBD, some problematic cases had high activity interquartile range and interdaily stability, and lower intradaily variability, reflecting a more preserved day-night contrast in activity levels, resembling the more consolidated rest-activity profile typically observed in PD without RBD. These cases also showed a lower magnitude of night movements, as can also be observed in supplementary figure 10, potentially contributing to reduced class separability.

Supplementary Figure 6. Three RLS cases that were the hardest for the PAT model classification. The 2 first days were chosen for visualization.

Supplementary Figure 7. Distribution of actigraphy-derived features in problematic cases with Restless Legs Syndrome (RLS). Colored dots represent individual cases identified as problematic by the classification model. Visual comparison of individual cases against the overall feature distributions highlights deviations in each metric that may contribute to model misclassification. Metrics where problematic cases had the greatest deviation from the median were chosen to display.

Supplementary Figure 8. Three PD cases without RBD that were the hardest for the PAT model classification. The 2 first days were chosen for visualization.

Supplementary Figure 9. Distribution of actigraphy-derived features in problematic cases with PD without RBD. Colored dots represent individual cases identified as problematic by the classification model. Visual comparison of individual cases against the overall feature distributions highlights deviations in each metric that may contribute to model misclassification. Metrics where problematic cases had the greatest deviation from the median were chosen to display.

Supplementary Figure 10. Three PD cases with RBD that were the hardest for the PAT model classification. The 2 first days were chosen for visualization.

Supplementary Figure 11. Distribution of actigraphy-derived features in problematic cases with PD with RBD. Colored dots represent individual cases identified as problematic by the classification model. Visual comparison of individual cases against the overall feature distributions highlights deviations in each metric that may contribute to model misclassification. Metrics where problematic cases had the greatest deviation from the median were chosen to display.

**PAT Model Explainability**

We performed an attention-based analysis of the PAT model, to gain insight into which temporal segments of the actigraphy signal contributed most strongly to model predictions. Specifically, attention weights from the final layers were extracted for representative samples from each cohort (RLS controls, PD-noRBD, and PD-RBD) in both classification tasks.

The resulting attention maps were projected onto the corresponding 24-hour activity profiles to identify time periods with higher relative contribution to classification decisions, as shown in Supplementary Figure 12. Across groups, the PAT model consistently assigned greater attention to nocturnal periods, suggesting that nighttime motor activity patterns were an important discriminative feature, as expected. This effect was most pronounced in PD-RBD cases, in which elevated attention weights were observed during periods of increased nocturnal movement activity.

Additionally, moderate attention peaks were observed during early afternoon periods in PD-RBD and, to a lesser extent, PD-noRBD samples. These time segments may correspond to diurnal rest or napping behavior.

It should be noted that attention weights reflect model-internal relevance and do not directly correspond to clinically interpretable circadian or sleep-related metrics. Therefore, these visualizations provide qualitative insight into the temporal regions influencing classification but should be interpreted cautiously in terms of physiological mechanisms.

Supplementary Figure 12. PAT model attention maps over 24-hour actigraphy profiles across diagnostic groups. Representative 24-hour mean activity profiles (black line; counts/min) from (a) RLS, (b) PD without RBD, and (c) PD with RBD. The heatmap reflects the relative importance assigned by the model to different time points during classification – RLS vs PD in a) and PD-RBD vs PD-noRBD in b) and c) – , based on its attention scores. These scores represent how strongly each segment of the 24-hour signal contributed to the model’s prediction. Values were normalized and projected onto a color scale, where red indicates time periods with greater influence on the decision and blue indicates lower influence.
